# Encoder-Decoder Architecture for Ultrasound IMC Segmentation and cIMT Measurement

**DOI:** 10.3390/s21206839

**Published:** 2021-10-14

**Authors:** Aisha Al-Mohannadi, Somaya Al-Maadeed, Omar Elharrouss, Kishor Kumar Sadasivuni

**Affiliations:** 1 Department of Computer Science and Engineering, Qatar University, Doha P.O. Box 2713, Qatar; aishaalmohannadi@outlook.com (A.A.-M.); elharrouss.omar@gmail.com (O.E.); 2 Center for Advanced Materials, Qatar University, Doha P.O. Box 2713, Qatar; kishorkumars@qu.edu.qa

**Keywords:** carotid intima-media thickness, IMT, CCA, segmentation, deep learning, encoder-decoder model

## Abstract

Cardiovascular diseases (CVDs) have shown a huge impact on the number of deaths in the world. Thus, common carotid artery (CCA) segmentation and intima-media thickness (IMT) measurements have been significantly implemented to perform early diagnosis of CVDs by analyzing IMT features. Using computer vision algorithms on CCA images is not widely used for this type of diagnosis, due to the complexity and the lack of dataset to do it. The advancement of deep learning techniques has made accurate early diagnosis from images possible. In this paper, a deep-learning-based approach is proposed to apply semantic segmentation for intima-media complex (IMC) and to calculate the cIMT measurement. In order to overcome the lack of large-scale datasets, an encoder-decoder-based model is proposed using multi-image inputs that can help achieve good learning for the model using different features. The obtained results were evaluated using different image segmentation metrics which demonstrate the effectiveness of the proposed architecture. In addition, IMT thickness is computed, and the experiment showed that the proposed model is robust and fully automated compared to the state-of-the-art work.

## 1. Introduction

The heart is an essential organ in the body, where its main job is to push the blood all around the human body. Furthermore, it is the main and central part of the cardiovascular system, which contains the blood vessels that form the blood circulation [1]. Moreover, cardiovascular diseases (CVDs) play a great role in the worldwide death toll, and this highlights the importance of early diagnosis of such disease. According to World Health Organization (WHO), CVD is the first cause of death in the world, taking 17.9 million lives each year [2].

According to the authors in [3], CVD is an abnormal illness that affects the heart and the blood vessels. With that being said, the authors in [4] highlighted that in their study of the worldwide deaths that were caused by CVDs, almost half of the deaths (48.5%) were associated with coronary heart disease, while strokes only took part in 20.8% of the population tested and the rest is for other diseases. Hence, it indicates the importance of preventing the progression of coronary heart disease.

**Figure 1:**
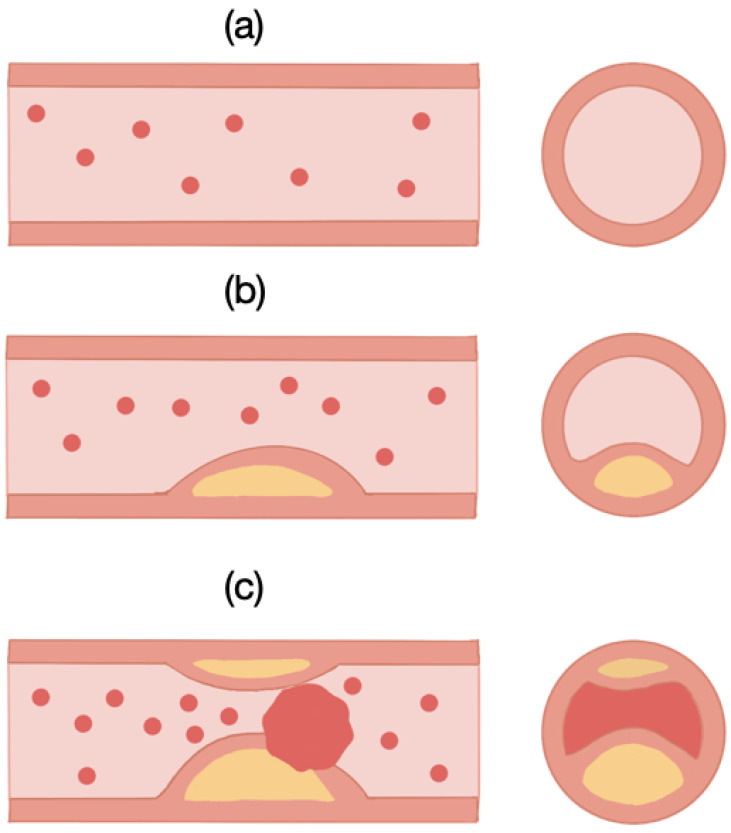
(**a**) Health artery, (**b**) formation of plaques in CCA, (**c**) atherosclerosis.

In addition, some of the risk factors of CVDs could be due to high blood pressure or high cholesterol. As a result, a buildup of inflammatory cells known as plaques in the artery wall, resulting in blood limitation to the heart and lower oxygen intake, can be one of the main causes of such disease. This phenomenon is known as atherosclerosis. As a result, early detection of this condition could aid in reducing the advancement of atherosclerosis as well as heart failure. In Figure 1, the plaque buildup is usually seen in the common carotid artery (CCA) and the internal carotid artery (ICA).

The carotid artery, which is made up of two blood vessels and has numerous components, including the internal, exterior, and common parts, is one approach to discover plaques in the arterial wall. Plaques can form in the interior segment of the carotid artery as well as the common blood vessels. Hence, plaques thicken the walls of these vessels, which is quantified as intima-media thickness (IMT). Thus, the difference between the lumen-initima (LI) and media adventitia (MA) walls can be measured to determine cIMT as a risk marker for early detection of heart disease [5]. Referring to a review done in [6], cIMT measures have shown to be able to predict CVD events independently of other risk variables; in fact, according to a study published in [7], it is a stronger predictor of strokes than other vascular disorders.

The carotid IMT test is a method of detecting IMT and diagnosing atherosclerosis that is carried out in clinics using an ultrasound instrument and is mostly performed by doctors. When the ultrasound image is obtained, the physician segments the IMT measurements manually. Another option is semi-automatic detection and segmentation, in which a physician locates the area of interest followed by automated segmentation of the artery walls. Furthermore, fully automated systems can detect and calculate cIMT without the need for a physician’s intervention. This highlights one challenge: systems must be accurate in their calculations in order to provide a reasonable evaluation of the IMT measurement. When a fully automated model is implemented, it eliminates the need for physicians. Hence, it encourages the employment of portable devices.

Furthermore, detecting IMC and measuring it have proven difficult in some cases, where locating the artery walls and determining its boundaries can vary depending on the quality of the B-mode ultrasound images. Furthermore, ground truth points generated by physicians may contain some errors due to differences in inter and intra-observer readings.

Using deep learning techniques to diagnose such disease can be beneficial in many ways, namely, it can be deployed in portable devices, hence, help patients in self-diagnosing themselves. Additionally, it can reduce the load on doctors that might be examining and diagnosing each patient including the ones with no risks. Many applications have been conducted for cIMT segmentation and identification using deep learning and machine learning techniques, however, the accuracy of the cIMT estimation is arguable.

In this paper, we focus more on evaluating the encoder-decoder model on IMC segmentation along with finding the best hyper-parameters for the model. This is mainly done using encoder-decoder networks that aim to compress the data to a latent representation and decode it using another decoder network to decompress the image, where latent representation commonly contains the features of the image. Additionally, we train and test the model using the encoder-decoder architecture as well as a dataset from [8] with pre-processing and post-processing techniques. The main aim of this research is to perform segmentation of B-mode ultrasound images using deep learning encoder-decoder architecture.

In this work, the main purpose is to develop a system that is able to segment the IMT in the arterial walls using deep learning models. Thus, deep learning models, specifically encoder-decoder models are investigated. The main contributions of the research are summarized as the following,

- Provide a comprehensive review of convolutional autoencoder (CAE) applications as well as IMT segmentation applications.
- Develop a convolutional autoencoder model for carotid intima-media complex (IMC) segmentation and IMT measurement on B-mode ultrasound images.
- Evaluate the effectiveness of CAEs in variation with hyper-parameters.
- Find an optimal architecture for CAEs by comparing the effectiveness of models with state-of-the-art methods.

In addition, we focus on main research questions to be able to evaluate the outcome of our solution, such as, how does the encoder-decoder model improve carotid IMT segmentation as well as how is it unique from previous solutions. Finally, we examine if the encoder-decoder model is able to be effective with the data augmentation on the given dataset with a limited number of images.

The sections for the rest of the paper are divided as follows; in Section 2, we highlight the recent work applied for carotid IMT segmentation and classification, including the encoder-decoder applications as well. Then, in Section 3, we propose our solution and present the model architecture for the deep learning model along with the data preparation process. Whereas, in Section 4, we present the experimental setup along with the evaluation metrics and the results of the model. After that, in Section 5, the results are discussed and the main challenges are pointed out. Finally, we conclude and explain the future work in Section 6.

## 2. Related Work

This section introduces the previous methods done for carotid IMT segmentation, as well as medical applications using encoder-decoder models [9], where there is a considerable amount of literature on carotid artery IMT segmentation using deep learning, machine learning, and contour techniques. The sections are structured as follows; the encoder-decoder applications in the medical field are introduced briefly. Then, the segmentation techniques for cIMT are tackled, followed by identification of the shortcomings of the current literature that has been implemented regarding this work.

### 2.1. Medical Encoder-Decoder Applications

Currently, encoder-decoder models are growing in the medical imaging field, as they consist of many types including merged techniques such as stacked autoencoders (SAE), stacked denoising autoencoders (SDAE), stacked sparse autoencoders (SSAE), and convolutional variational autoencoders (CVAE). Many studies have been published on CVAE including medical applications to predict post-trauma health outcomes [10]. Another study was done by the authors of [11], which included using CVAE to automatically detect plant diseases, as well, the authors in [12] developed CVAE based system for electrocardiographic imaging (ECGI).

Several studies have been conducted for encoder-decoder models in medical applications namely, mortality risk prediction [13], as well as chest radiology improvement using denoising autoencoders [14]. Regarding image segmentation in the medical field, encoder-decoder models show a huge impact on the accuracy of applications comparing to other models. Thus, the authors of [15] claimed that their application for 3D image segmentation using CT scans shows improvement in results.

### 2.2. Carotid IMT Segmentation Applications

Many attempts have been made regarding carotid IMT segmentation and classification, however, only a few show competitive results given the fact that IMT segmentation is the most sensitive step as the thickness measurements depend on the accuracy of the IMT segmentation. The authors of [16] use support vector machines in order to train and segment the carotid IMT. In their method, they used 49 ultrasound images and divided them into two sets with 50% for training and the rest for testing. Their method provided 93% accuracy, and as for the IMT measurement, they found it to be 0.66 mm.

One of the first attempts for carotid segmentation was done by Loizou et al. (2013) [17], where they implemented a semi-automated snake-based segmentation system proper for complete CCA segmentation. Their method concentrated on estimating IMT measurements by manually defining the carotid plaque and diameter and then applying the snake algorithm to get the measurements. The dataset that was used for this algorithm was 300 2D ultrasound images. Their method did not result in a significant difference from the state-of-the-art, as it was limited to only manual readings.

Furthermore, the authors of [18] attempted to implement a fully automated segmentation system using adaptive snake’s contour as well as level set segmentation. When comparing both techniques together the authors found out that the snake’s contour method outperformed the level set segmentation. Another technique was developed by the authors of [19] that also does not depend on AI, their method included bulb edge detection and then segmental IMT measures are applied according to the detected edge. Their dataset consisted of 649 images that have between moderate and heavy lighting. They got a significantly low error in calculating the IMT measurement which is around 0.0106 mm and precision of merit that equals 98.23%.

Another technique was built by the authors of [20] that avoided the implementation of deep learning for IMT segmentation. The authors illustrated that they used wind-driven optimization technique for carotid IMT segmentation, as they focused on developing a fully automated region of interest (ROI) extraction as well as they used for intima-media complex a threshold-based method. Their results included an IMT measurement of 0.69 mm as they claimed that their method outperformed other work in the literature.

Experiments on IMT segmentation were not limited to non-AI only, where authors in [21] implemented a screening tool that integrates a two-stage artificial intelligence model for IMT and carotid plaque measurements, which consists of a CNN and fully convolutional network (FCN). The system goes through two deep learning models, as the first divides the CCA from the ultrasound images into two categories the rectangular wall and non-wall patches. Then, the region of interest is analyzed and fed to the second stage, where they identify some features to calculate the carotid IMT and the plaque total as well. Furthermore, their dataset consisted of 250 images, whereas their results while using the proposed AI model showed an error of IMT measurement that equals 0.0935 mm.

As investigations of IMT segmentation went on with deep learning and machine learning, the authors of [22] proposed a method for segmentation using CNN. Therefore, the researchers applied an algorithm that finds the ROI using the CNN architecture which includes eight layers. Moreover, they trained the network using 220 left and right CCA images for ROI localization. After that, the intima-media complex area is extracted in order to measure the IMT. The mean difference for IMT measurement is found to be 0.08 mm, where they had an accuracy of 89.99% for the CNN network.

Another research group [23] investigated IMT segmentation in video interpretation of IMT measurement using CNN. They performed CNN using six layers, and they claimed that they were able to achieve a low error rate in their measurements as they got a result of 2.1 mm error with only one failure for testing subjects. Furthermore, another technique was used by Joseph and Sivaprakasam (2020) [24], where they used double line echo patterns coming from the B-mode and A-mode ultrasound images to identify both arterial walls. Their method showed an error of IMT measurement that equals 0.18 mm.

Another combined method was implemented by researchers in [25], where they used deep learning for IMT measurement for patients with diabetes. Their method includes two stages, the first is the CNN network that is used for segmentation and the other is machine learning-based regression. Therefore, their output was the borders of the lumen intima and the media-adventitia which is used to calculate the carotid IMT. In their work, they used a dataset of 396 B-mode ultrasound images, as they got the result of the error for cIMT measurement to be around 0.126 mm. Researchers claimed that their method was 20% improved compared to other non-deep learning methods.

One more deep learning method was discussed by researchers in [26], where they used CNN with multiple hidden layers for image classification. They were able to test the network using 501 ultrasound images dataset and achieve an accuracy of 89.1% for IMT classification. The other method was developed by the authors of [27], where they used four classification algorithms for IMT measurement, the algorithms consisted of SVM with linear kernel, SVM with radial basis kernel, AdaBoost, and random forest. They evaluated their method using a dataset that consisted of 29 images, and they concluded that the best results were for the integrated random forest method which results in 80.4% sensitivity and 96.5% specificity.

One study has been made regarding IMT measurements using autoencoders and this was done by the authors of [28], their method included ROI prediction and then lumen-intima interface (LII) and media-adventitia interface (MAI) walls predictions in the predicted ROI, as their dataset consisted of 67 images. The authors claimed that they used extreme learning machines (ELM) along with autoencoders in order to distinguish which block is included in the ROI and which is not. Whereas the LII and MAI recognition was done using pixel classification. Moreover, they evaluated their IMC segmentation by using accuracy, specificity, sensitivity, and Matthews correlation coefficient (MCC). Their results used sensitivity and specificity for evaluating ROI prediction, on the other hand, accuracy and MCC were used LII and MAI. The final results were a mean IMT measurement of 0.625 ± 0.1673 mm, with an accuracy for LII of 99.30% and for MAI of 98.8%, and the MCC for LII and MAI was 98.03% and 97.05%, respectively.

Given the above methods, one research used autoencoders for IMT measurement which is the one done in [28]. Their findings were done using machine learning and autoencoders for ROI localization only, where they used another technique for IMT segmentation and recognition. Moreover, some limitations were identified, such as the fact that using semi-automated systems could lower the feature of having a portable system, also some methods used clinical instruments that are not portable. Furthermore, when compare to non-AI methods, we observe that errors found can be lower and accuracy can be enhanced further when using AI methods. To illustrate more, we conclude that work done in [25] has a lower error calculated in IMT measurement (0.126 mm) than the one in [24], which is 0.18 mm. Therefore, in our research, we focus on implementing a solution that is fully automated and supports portability along with taking into account segmentation metrics.

One more thing to point out is that Table 1 shows the different applications that have been done for IMT predictions including AI and non-AI techniques. However, comparing these applications together may not be fair since each application uses a different set of datasets, and the percentage of the dataset for training that was used is not the same. With that being said, we can make relative comparisons where we can point out the outcomes of each application given their architecture or method used. Thus, in this work, we did not compare our results quantitatively with the provided literature since different datasets are used. Instead, a comparison is only done with applications [8].

## 3. Proposed Method

Carotid artery detection and segmentation can be one of the important solutions for healthcare using medical imaging techniques. The lack of labeled large-scale datasets is one of the challenges that can be faced for this task. In the case of the carotid artery, one dataset was found with no labeling. Due to the fact that segmentation of regions of a medical image is very helpful for doctors to diagnose, a pre-processing technique on the original dataset is used to prepare this dataset for segmentation purposes. Then, using the proposed deep-learning-based model the carotid artery is segmented. To remove the false segmented pixels in the images a post-processing technique is applied using morphological operation. In this section, each step is described in detail.

### 3.1. Data Preparation

The dataset used for this paper is a dataset in [8]. It contains 100 carotid IMT B-Mode ultrasound images with their ground truth points determined by two clinical experts. In their work, Loizou et al. [8] highlighted that images were taken from 42 female and 58 male symptomatic patients aged between 26 and 95, where they produced longitudinal ultrasound images.

The images were obtained from the ATL HDI–3000 scanner (Advanced Technology Laboratories, Seattle, WA, USA), and were logarithmically compressed to produce images with 768 × 576 pixels resolution and 256 grey levels. The scanner has a multi-element ultrasound scan head with an operating frequency range of 4–7 MHz, an acoustic aperture of 10 × 8 mm, and a transmission focal range of 0.8–11 cm, with 64 elements fine pitch high-resolution, and 38 mm broadband array. Furthermore, the bicubic method was used to resize digital images to a standard pixel density of 16.66 pixels/mm.

**Figure 2:**
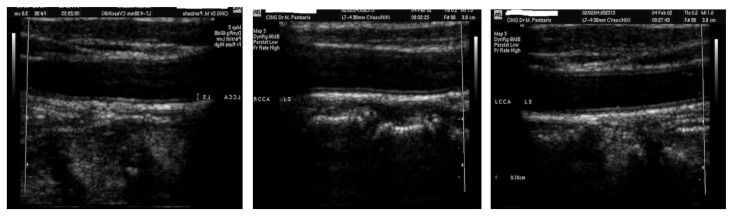
Three sample images from the dataset [8].

Figure 2 illustrates three sample images from the dataset that we work within this paper. Given that the ultrasound image is all that is required, the frames in the samples that included patient information were removed as part of the pre-processing step.

Similarly, the authors of [8] identified the IMT measurements from both experts with a number of techniques. They used speckle reduction, as well as normalization as pre-processing steps. In this research, we focus only on normalized images, hence, only IMT measurements for normalized images are used. Table 2 shows the expert’s measurements for IMT in the case of normalized 100 images.

The experts readings included two time periods one at time 0 months and the other at time 12 months. According to others in [8], this was done to test the intra-observer variability for the same expert. This means that the experts highlighted the carotid walls two times in different period of time for the same image in order to assess observer errors.

#### 3.1.1. Pre-Processing

First of all, we took the raw images and removed the frames that were not of interest. After that, images were normalized and taken to be processed using Sobel and Prewitt gradient methods that are available built-in functions in MATLAB. We have examined them with other filters like the canny filter, however, given that it is an edge detector, the IMT region was identified as small, disconnected circles. Thus, it was not retrieving the features accurately. Then, we experimented with gradient images and found that they were able to distinguish the IMT region properly. Both methods produced gradient magnitude as well as gradient direction. For this implementation, we stored only the normalized gradient direction for both methods. After trying other filters, the gradient directional images were found to be the most accurate shows the cIMT more clearly than the rest of the images.

**Figure 3:**
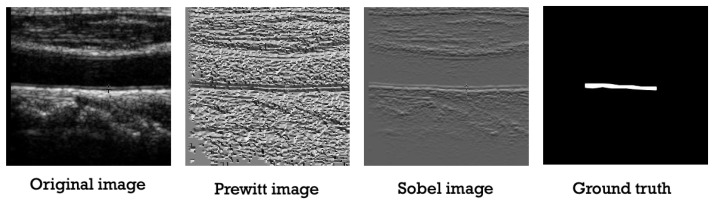
The pre-processing phase.

As for the ground truth points, we converted the points to binary images in order to input them as labels in the deep learning model, as well as to compare them with predicted images in the testing phase. In Figure 3, the original image along with the gradient images and the produced ground truth image are shown. Additionally, in order to train the model with data augmentation, the newly generated ground truth mask images were produced using lines that connects the ground truth manual points given in the dataset without using a threshold.

#### 3.1.2. Data Augmentation

Data augmentation is mainly used when we have a small dataset and would like to increase the number of images in a given dataset [29]. Thus, it provides small operations that can give the ability to rotate, flip, shift, zoom, or translate a given image without changing its content. Hence, we keep the final image as the original image features. Moreover, in order to do data augmentation, we need to have binary mask images since we are changing the display of an image, then the given mask should go through the same process.

In this stage, we use special features to implement augmentation namely, rotation, width and height shift, and zoom. Table 3 shows the values used for the augmentation. The augmentation was done using ImageDataGenerator library in python, where it was used to augment both image and its binary mask.

### 3.2. Encoder-Decoder Architecture

The introduction of deep learning techniques, such as convolutional neural networks, improved the image segmentation task in terms of the quality and quantity of segmented parts in an image. From the famous architecture that used CNNs layers for images segmentation, we can find SegNet [30] and U-Net [31] which are encoder-decoder-based models that achieved good results in semantic segmentation. Both architectures are capable of binary and multi-class segmentation, where binary image segmentation is much easier than colored image segmentation. Thus, we were inspired by the SegNet architecture to implement the proposed model for segmenting the carotid artery from ultrasound images. The proposed architecture used two inputs instead of the model’s single input as used by U-Net and SegNet. A model’s multiple input can assist in the extraction of useful information while allowing for multi-feature learning. The proposed model includes two encoders for feature extraction, a fusion layer, a decoder with upsampling, and convolutional layers to produce the final results.

**Figure 4:**
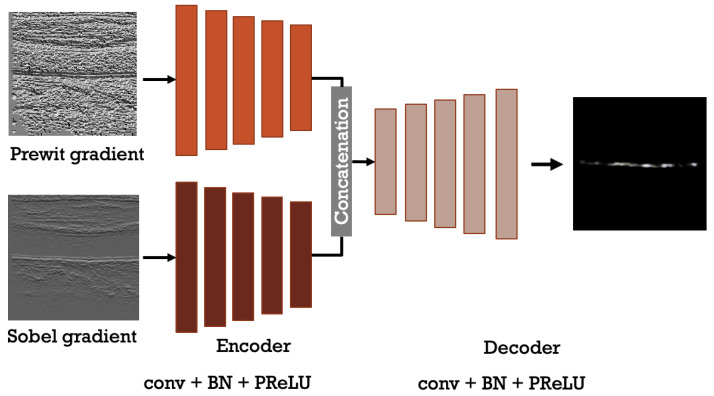
The model architecture used for the research solution.

Each encoder is implemented based on VGG-19 [32] backbone as SegNet, which is a series of (conv+BN+PReLU) layers with pooling layers and batch normalization (BN) [33]. The two encoders are merged by concatenating the feature maps of each encoder output. Like SegNet and U-Net, the proposed decoder employs blocks of upsampling (unpooling) and convolutional layers (upsampling+Conv+BN+PReLU). The encoder part’s feature maps are converted into the final label by the decoder, which takes into account spatial restoration. The same structure used in the encoder is also used for the decoder by replacing the pooling layers with upsampling layers. Hence, the final architecture is shown in Figure 4. For the loss function, the SoftMax function is used to calculate the loss function:

(1) $$loss=\frac{1}{N}\sum_{N=1}^{N}\sum_{i=1}^{k}y_{j}^{i}log\left(\frac{exp(p_{j}^{i})}{\sum_{l=1}^{k}exp\left(p_{j}^{l}\right)}\right)$$

where *N* is the number of pixels in the input image, *k* is the number of classes and, for a specified pixel *i*; $y_{i}$ denotes its label and the prediction vector.

For this paper, we use two components namely, the Sobel gradient direction image and Prewitt gradient direction image as inputs of the model. Additionally, these images went through pre-processing steps which are discussed in Section 3.1.1.

In the case of the segmentation process, it was mainly done by using 80% of the final dataset and converting it to both Sobel gradient and Prewitt gradient. These images were fed to the encoder-decoder architecture described in Figure 4. During the training phase, we trained the model multiple times in order to get the best performance and tune the hyper-parameters for better accuracy. Finally, the training was done using 50 epochs along with 10 steps per epoch, which means it increases the data augmentation for each epoch 10 times.

Furthermore, post-processing techniques were applied since the final segmented image had some noise that needed to be reduced and specific regions of interest (ROI) needed to be highlighted. For that, we used morphological opening, which removes any small noise in the image and can detect discontinued blobs. Additionally, we used morphological closing in order to avoid having a discontinued segmentation of the carotid artery IMT. The shape for the morphological operations was a rectangular shape with different sizes each time. Firstly, morphological close is applied with a size of [(2 ,30] in order to close the gaps between discontinued IMT. Then, morphological open with a size of [2 ,30] is applied to remove small noises from the image. Finally, morphological close is applied with a size of [3 ,30].

## 4. Experimental Results

This section tackles the setup and evaluation metrics that lead to the given results as well as an analysis of the produced results.

### 4.1. Experimental Setup

In order to implement the architecture and train it using the model described in Section 3.2, we use python programming language. We built and trained the model on NVIDIA GeForce GPU using Python. The implementation was done using Windows 10 operating system and the framework was done on Anaconda. Python version 2.7 was used for the training process. Keras and Tensorflow software packages were used to model and evaluate the results.

### 4.2. Evaluation Metrics

The evaluation of the deep learning model performance computed in the testing phase was based on the segmentation metrics [34,35]. These metrics are defined as follows:

- **Precision**: This calculates how close the values are to each other and how close they are to the true values.
- **Recall**: Also known as sensitivity. This is the ratio of the correct results by the overall correct data.
- **F1 Measure**: This is calculated using both precision and recall, where it gives an overall overview of the performance of the system.
  (2) $$F1\;Measure=\frac{2*Precision*Recall}{Precision+Recall}$$
- **Sorensen Dice Coefficient**: This calculates the similarity of two samples and is mainly used to validate image segmentation algorithms. It is also more about the percentage of overlap between two images.
  (3) $$Dice=\frac{2*TP}{2*TP+FP+FN}$$
- **Jaccard Index**: This is the percentage of similarity for two images. It is similar to the Dice index, however, the Jaccard index takes into account true positive only once, while in the Dice Coefficient it does it twice.
  (4) $$Jaccard\;Index=\frac{TP}{TP+FP+FN}$$

We focus mainly about the F1 measure, Dice coefficient, and the Jaccard index in this work, as they mainly evaluate the similarity and the efficiency of the model and segmentation algorithm.

### 4.3. Evaluation

During the implementation of the deep learning model, we did extensive training as we ran the experiments various times with different numbers of epochs. This included changing the batch size as well as experimenting with the input images along with the pre-processing techniques.

Firstly, the training was done on the Prewitt and Sobel images as inputs with batch sizes equal to 32 and 8 as well as we included data augmentation in this phase. As a result, it was clear that the 32 batch size was not segmenting only the desired part and 8 batch size was more accurate. Thus, we trained the model using 8 batch size and augmentation in the second phase. Furthermore, we examined with changing input images as Prewitt and Sobel, and in the other experiment we made the inputs as the original image and Sobel image. However, the two gradient images were giving more accurate results. The first results were done using the architecture without batch normalization layers. We examined then the batch normalization layers on the final results and it gave preferable results than the outputs previously examined.

Similarly, Table 4 illustrates the trials that were done and how the parameters were changed. The last two trials included the batch normalization layer, which had the higher percentages in the final results explained in Table 5. In addition, in Table 6 the performance of each trial is provided.

**Figure 5:**
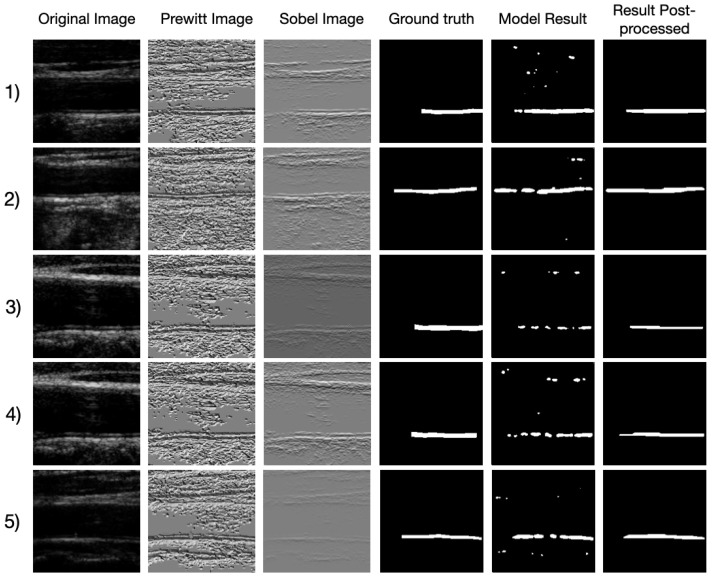
The results of five images for the final training.

After tuning the hyper-parameters and decreasing the learning rate we were able to get the results shown in Figure 5.

In addition, the results show better similarity with the ground truth with little extension of the line. During the testing phase, we evaluate the model using three metrics discussed in Section 4.2. The results of the metrics are shown in Table 6.

According to the Jaccard index and the Dice coefficient, they show a similarity of the tested data with the binary masks. The highest percentage is the F1 measure, where it gives an overview of the performance of the system.

The results showed that the system has somewhat good performance, however, it can be further enhanced, where pre-processing or post-processing techniques need to be further enhanced. Results might not give the best accuracy due to the fact that the dataset is not very clean, as it was hard to work with.

Moreover, we performed pixel calculations to get the thickness of the predicted IMT measurement. The calculations were made by calculating the distance from the upper boundary to the lower boundary. It was done using MATLAB functions bwdist(), as it calculates the vertical distance of a binary object. Additionally, the local max value was taken and then the mean value was calculated for all tested images to get the thickness as 2.989 pixels. Furthermore, converting pixels to mm we get 0.54 mm as the mean IMT measurement.

In comparison to the work done in [8], as well as the ground truth, Table 7 illustrates the error found in both the dataset and the proposed method compared to the ground truth. As explained before in Table 2, the ground truth IMT has been determined by two experts at a certain time. We observed from the table that the minimum IMT measurement in the proposed solution is smaller than the ground truth. This is due to some predictions where the IMT was not clear in the image, thus, the model was only able to distinguish a small part of the IMT. Furthermore, the median value of the proposed model is close to the reading for Expert 2 than the one in [8]. Regarding the max value, the proposed model was also able to achieve a similar thickness as Expert 2. With that being pointed out, the dataset was used in [8] had a semi-automated model, comparing our model to theirs, our results were better given the automation.

## 5. Discussion and Challenges

Given the results discussed in Section 4, we were able to achieve a segmented region for the carotid IMT, which was then used to estimate the thickness. For this paper, we were able to train and test the images and compare them to the ground truth points.

During the implementation of this solution, many other architectures were investigated, including UNet segmentation using MATLAB. These models were trained using more than 50 epochs with no good results. Therefore, the encoder-decoder architecture was able to produce segmented output which achieved a good performance. The results of this model look promising and are good for future expansion.

One of the main challenges faced in this research is finding a good segmented and annotated dataset. We faced many issues to get a dataset and we were able to receive the dataset that we worked on. Moreover, the dataset was not clean enough to be processed, hence, it was time consuming to work on these images, where in some cases the IMT was not very clear. Thus, the output for these images from the model are discontinued parts of sections around the IMT. Additionally, the dataset has no recent studies, which makes it hard to compare between results.

According to the research questions described in Section 1, we can conclude now that the model was able to segment IMC fairly well. However, due to the lack of variety of images in the given dataset, it is not clear if the model can improve IMT segmentation. Thus, further research needs to be done regarding the dataset. Moving to the second question, the model chosen has not been used before for IMT segmentation and it has shown good results for this dataset and it is open for further improvements. We also observed from the trials and experimentation that 8 batch output with augmentation showed better segmentation than the one without augmentation. Hence, the data augmentation was effective on the dataset along with the encoder-decoder model.

In general, after comparing with the results found in [8], we identify that the proposed method is robust and fast and is fully automated compared to their semi-automated snake segmentation.

## 6. Conclusions and Future Direction

In conclusion, CVDs take millions of lives on a yearly basis, which means it is important to provide people with ways for early diagnosis of such a disease. Many implementations were done for such a problem using computer vision techniques for B-mode ultrasound images. In addition, we looked at recent work on carotid intima-media thickness segmentation and encoder-decoder applications. In this research, we investigated a deep learning model, specifically a convolutional autoencoder with two inputs for two encoders, and identified the optimum hyper-parameters and architecture that produced results that were similar to the dataset’s provided ground truth. We trained the encoder-decoder architecture using 10 steps per epoch and 50 epochs and 80% of the dataset. We were able to obtain results of 79.92%, 74.23%, and 60.24% for the F1 Measure, Dice coefficient, and Jaccard index, respectively. We also calculated the IMT thickness, which was 0.54 mm. The model showed good performance with the lowest error of 0.03 mm, compared to the ground truth data.

Further enhancement could be done by experimenting with the optimized model along with other ultrasound B-mode carotid datasets, this would give an overview of the generality of such system and the performance given other images. Furthermore, we could experiment with different modern filters to input with the model and evaluate the performance. Our proposed system is highly recommended to be used along with a portable device that acquires ultrasound images and processes them in order to give patients the ability to early diagnose themselves.

## Figures and Tables

**Table 1:** Literature summary of cIMT segmentation applications.

Method	Dataset Size	Techniques	Metrics	Error in IMT (mm)
[16] (2018)	49	SVM	the correlation coefficient R, accuracy	0.01
[17] (2013)	300	snake’s segmentation	Wilcoxon-sum test	0.01
[18] (2012)	100	snake’s contour, level set segmentation	Wilcoxon-sum test	IMT SC: 0.12, LS: 0.09
[19] (2017)	D1: 172, D2: 649	bulb edge detection	precision accuracy, sensitivity, specificity	0.01603 ± 0.0031
[20] (2018)	D1: 100, D2: 25	wind driven optimization technique	The correlation coefficient R	-
[21] (2020)	250	CNN, FCN	correlation coefficient, Polyline distance metric (PDM),accuracy	0.0935 ± 0.0637
[22] (2018)	220	CNN	accuracy	0.08
[23] (2016)	92 videos	CNN	-	2.1
[24] (2020)	40	dignal processing	accuracy of RF frame sequqences with different SNR	0.18
[25] (2018)	396	FCN, CNN, regression	PDM, Precision of Merit (PoM)	0.126 ± 0.134
[26] (2019)	501	CNN	precision, recall, f1score, support	-
[27] (2017)	29	SVM	specificity, sensitivity, dice coefficient	-
[28] (2016)	67	ELM autoencoder	accuracy, specificity, sensitivity, Matthews correlation coefficient	0.1673

**Table 2:** Ground truth measurements for IMT in mm.

	Time 0	Time 12	Time 0	Time 12	Time 0	Time 12	Time 0	Time 12
**Expert**	**Mean (Std)**	**Mean (Std)**	**Min (Std)**	**Min (Std)**	**Max (Std)**	**Max (Std)**	**Median (Std)**	**Median (Std)**
1	0.68 (0.17)	0.68 (0.17)	0.52 (0.15)	0.52 (0.15)	0.85 (0.21)	0.85 (0.21)	0.66 (0.18)	0.66 (0.18)
2	0.61 (0.17)	0.57 (0.13)	0.54 (0.14)	0.47 (0.14)	0.7 (0.2)	0.66 (0.14)	0.61 (0.17)	0.61 (0.14)

**Table 3:** Data augmentation parameters.

Feature	Value
Rotation	10
Width shift	0.2
Height shift	0.2
Zoom	0.2

**Table 4:** Experimental trials for hyper-parameter tuning.

Trial	Input 1	Input 2	Batch Size	Epochs	Steps/Epoch	Learning Rate	Augmentation
1	Prewitt	Sobel	32	50	-	0.0001	No
2	Prewitt	Sobel	8	50	-	0.0001	No
3	Prewitt	Sobel	8	15	5	0.0001	Yes
4	Original	Sobel	8	5	50	0.0001	Yes
5	Prewitt	Sobel	8	7	80	0.00001	Yes
6	**Prewitt**	**Sobel**	**8**	**50**	**10**	**0.00001**	**Yes**

**Table 5:** Evaluation of the experimental trials.

Trial	Input 1	Input 2	Batch Size	F1 Measure	Jaccard Index	Dice Coefficient
1	Prewitt	Sobel	32	-	50.77%	36.93%
2	Prewitt	Sobel	8	64.92%	45.65%	60.44%
3	Prewitt	Sobel	8	70.77%	45.43%	60.51%
4	Original	Sobel	8	67.63%	46.64%	61.31%
5	Prewitt	Sobel	8	73.63%	52.29%	66.07%
6	**Prewitt**	**Sobel**	**8**	**79.92%**	**60.24%**	**74.23%**

**Table 6:** Improved model architecture results.

Metric	Proposed Model
F1 Measure	79.92%
Precision	81.18%
Recall	82.06%
Dice Coefficient	74.23%
Jaccard Index	60.24%

**Table 7:** Comparison of the results.

	Expert 1	Expert 2	Snake’s Segmentation [8]	Proposed Solution
Normalized mean IMT measurement(mm) (std)	at time 0,12: 0.6 8 (0.17)	at time 0,12: 0.6 1 (0.17), 0.5 7 (0.13)	0.6 7 (0.13)	0.54
Error in mean IMT	-	-	Expert 1: at time 0,12: **0.01**, Expert 2: at time 0,12: **0.06,0.1**	Expert 1: at time 0,12: **0.14**, Expert 2: at time 0,12: **0.07,0.03**
Normalized IMT min(std)	at time 0,12: 0.5 2 (0.15)	at time 0,12: 0.54 (0.14), 0.47 (0.14)	0.51 (0.14)	0.18
Normalized IMT max(std)	at time 0,12: 0.8 5 (0.21)	at time 0,12: 0.7 (0.2), 0.66 (0.14)	0.86 (0.17)	0.71 (0.13)
Normalized IMT median(std)	at time 0,12: 0.6 6 (0.18)	at time 0,12: 0.61 (0.17), 0.61 (0.14)	0.66 (0.12)	0.60 (0.14)

## Data Availability

Not applicable.
